# Crystal structure of [1,2-bis­(di­phenyl­phosphan­yl)benzene]­hepta­carbonyldi-μ-hydrido-(μ_3_-2,4,6-tri­methyl­phenyl­phosphin­idene)-*triangulo*-triruthenium

**DOI:** 10.1107/S2056989017007770

**Published:** 2017-06-02

**Authors:** Taeko Kakizawa

**Affiliations:** a Department of Chemistry and Biochemistry, School of Advanced Science and Engineering, Waseda University, Shinjuku, Tokyo 169-8555, Japan

**Keywords:** crystal structure, cluster, phosphine, ruthenium

## Abstract

The title trinuclear ruthenium cluster, [Ru_3_(C_30_H_24_P_2_)(C_9_H_11_P)(CO)_7_(μ-H)_2_], has a triangular Ru_3_ core that is capped with a mesitylphosphin­idene ligand, μ_3_-PMes (Mes = mesityl = 2,4,6-tri­methyl­phen­yl). The 1,2-bis­(di­phenyl­phosphan­yl)benzene mol­ecule acts as a bidentate phosphine ligand *via* two P atoms connecting to a single Ru atom. The title compound crystallizes with two independent mol­ecules in the asymmetric unit.

## Abstract 2

The title compound, [Ru_3_(C_30_H_24_P_2_)(C_9_H_11_P)(CO)_7_(μ-H)_2_], crystallizes with two independent mol­ecules in the asymmetric unit, which have a similar conformation. The mol­ecules have a trigonal–pyramidal structure of the phosphin­idene-capped triruthenium core with the bidentate phosphine ligand coordinating to one Ru atom.

## Chemical context   

In previous reports for cluster syntheses, bidentate phosphines occasionally act as spacer ligands that connect two cluster units to build up large clusters. For example, we have reported the successful synthesis of [Ru_3_(CO)_8_(μ-H)_2_(μ_3_-PMes)]_2_(μ-diphosphine) (Mes = mesityl = 2,4,6-tri­methyl­phen­yl) (Kakizawa *et al.*, 2015 ▸) by the linking of two phosphin­idene-capped Ru_3_ clusters formulated as Ru_3_(CO)_9_(μ-H)_2_(μ_3_-PMes) (Kakizawa *et al.*, 2006 ▸) with chelating diphosphine moieties such as 1,2-bis­(di­phenyl­phosphan­yl)ethane by thermal reaction. In the case of BDP [1,2-bis­(di­phenyl­phosphan­yl)benzene], the linking of two Ru_3_ units did not occur, and the title triangular–pyramidal cluster, Ru_3_(μ-BDP)(CO)_7_(μ-H)_2_(μ_3_-PMes), was obtained.
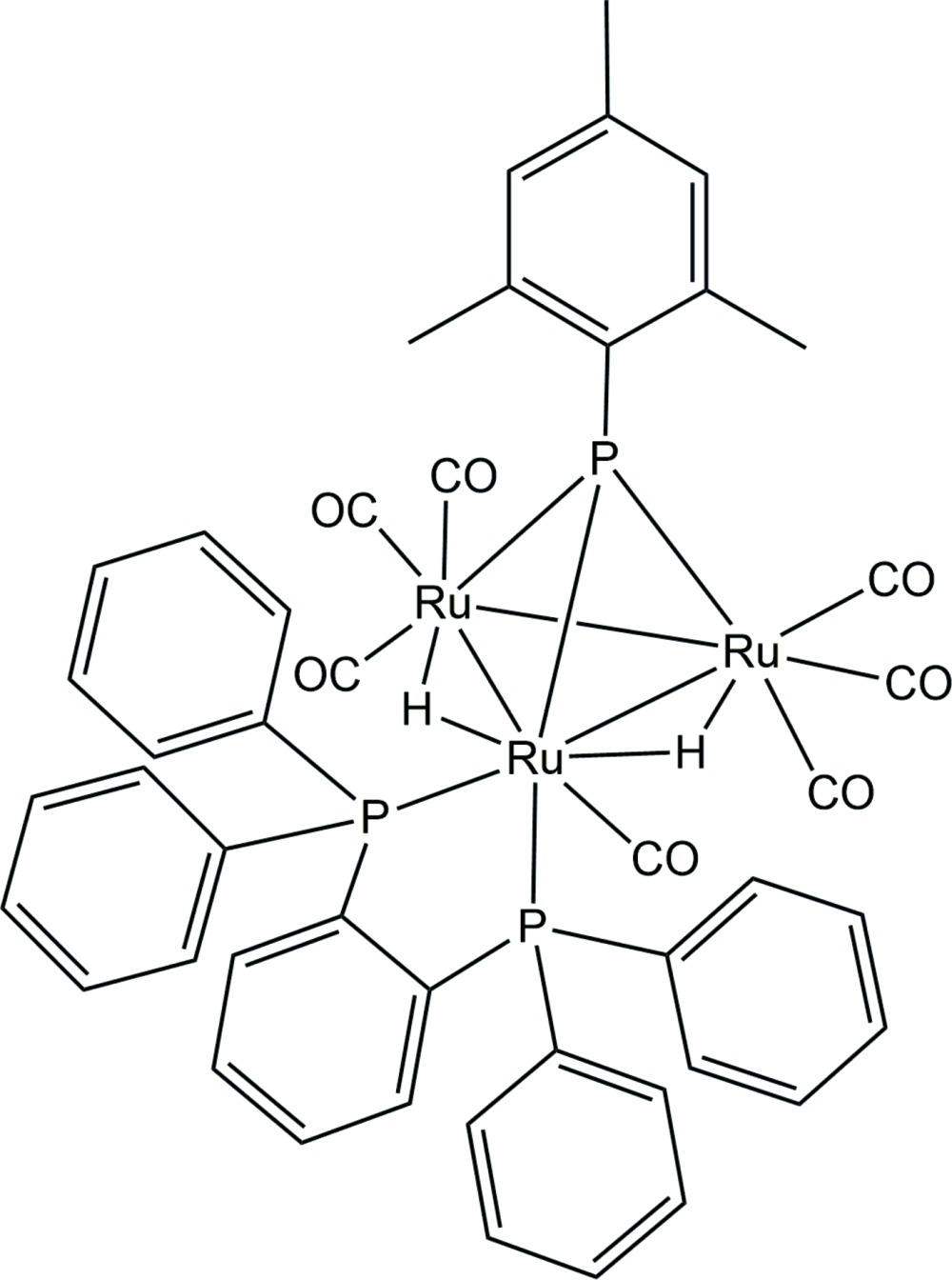


## Structural commentary   

**Figure 1:**
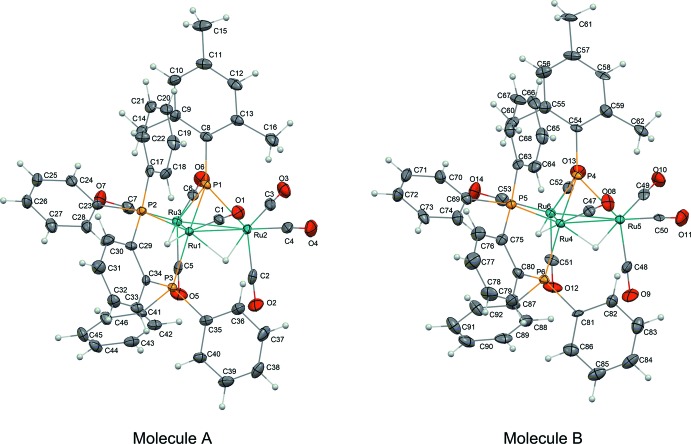
The structures of the two independent mol­ecules *A* and *B* of the title compound. Displacement ellipsoids are drawn at the 50% probability level for non-H atoms.

The title compound crystallizes with two independent mol­ecules *A* and *B* (Fig. 1 ▸), which show quite similar conformations to each other. The mol­ecules have a trigonal–pyramidal structure of the phosphin­idene-capped triruthenium core. One CO ligand and two phospho­rus atoms of BDP coordinate to a single Ru atom (Ru1 for molecule *A* and Ru4 for molecule *B*). In the triangular Ru_3_ moiety, the Ru1—Ru2, Ru1—Ru3 and Ru2—Ru3 bond lengths are 2.9297 (8), 3.0089 (8) and 2.7945 (8) Å, respectively, for mol­ecule *A*, and Ru4—Ru5, Ru4—Ru6 and Ru5—Ru6 are 2.9220 (8), 3.0018 (8) and 2.7902 (8) Å, respectively, for mol­ecule *B*. The longest bond lengths are for Ru1—Ru3 and Ru4—Ru6 in the two mol­ecules and might be caused by steric repulsion between the phenyl groups of BDP, the mesityl group on the phosphin­idene ligand, and the carbonyl groups. The coordin­ating BDP moiety shows a distorted five-membered ring with the Ru1—P2—C29—C34 and Ru1—P3—C34—C29 torsion angles being −13.9 (6) and 19.4 (6)°, respectively, for mol­ecule *A*, and Ru4—P5—C75—C80 and Ru4—P6—C80—C75 being −14.7 (6) and 21.8 (6)°, respectively, for mol­ecule *B*.

## Supra­molecular features   

**Figure 2:**
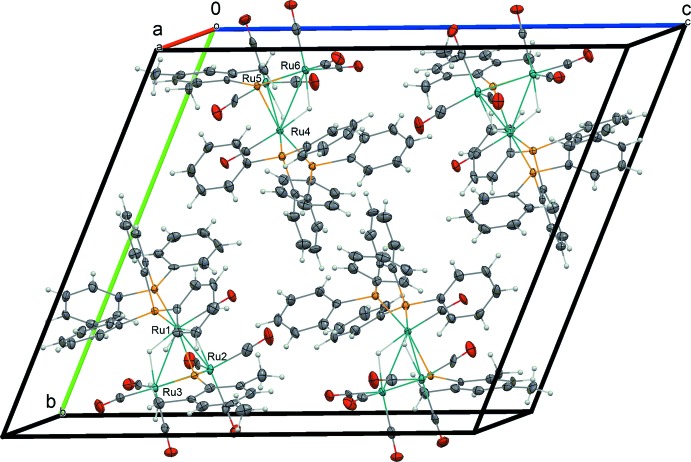
A packing diagram of the title compound, viewed along the *a* axis. Displacement ellipsoids are drawn at the 50% probability level for non-H atoms.

The packing of the title compound is shown in Fig. 2 ▸. No significant C—H⋯π or π–π inter­actions are observed within each independent mol­ecule or between adjacent mol­ecules.

## Database survey   

The crystal structures of similar coordination modes of BDP in which two phospho­rus atoms connect to one Ru atom in the polynuclear clusters have been observed, *i.e.*, HRu_6_(μ_5_-C)(μ_3_-P)(CO)_14_(BDP) (Watson *et al.*, 2007 ▸), 1,1-H_4_Ru_4_(CO)_10_(BDP) (Nesterov *et al.*, 2007 ▸), and the cationic trinuclear ruthenium complex [Ru_3_(μ_2_-Cl)_3_(μ_3_-Cl)_2_(BDP)_3_]PF_6_ (Mashima *et al.*, 1997 ▸). Mononuclear ruthenium complexes with BDP have also been reported, *i.e.*, Ru(CO)_3_(BDP) (Bunten *et al.*, 2000 ▸), [CpRu(PPh_3_)(BDP)]Cl (Guan *et al.*, 2003 ▸), CpRu(BDP)H (Guan *et al.*, 2003 ▸), [RuCl(BDP)(*cis*-1,3,5-tri­amino­cyclohex­ane)]Cl (Gamble *et al.*, 2013 ▸), [Ru(2,2′:6′,2′′-terpyrid­ine)(BDP)(CH_3_CN)](PF_6_)_2_ (Nakamura *et al.*, 2014 ▸), [Ru(2,2′:6′,2′′-terpyridine))(BDP)(NO_2_)](PF_6_) (Nakamura *et al.*, 2015 ▸) and Cp*Ru(BDP)(PPh_2_) (Sues *et al.*, 2014 ▸)**.**

## Synthesis and crystallization   

The title compound was synthesized following a literature procedure (Kakizawa *et al.*, 2015 ▸) with Ru_3_(CO)_9_(μ-H)_2_(μ_3_-PMes) (Kakizawa *et al.*, 2006 ▸) and 1,2-bis­(di­phenyl­phosphan­yl)benzene in a 2:1 molar ratio. Purification of the reaction mixture with silica-gel chromatography gave the title compound in high yield along with unreacted Ru_3_(CO)_9_(μ-H)_2_(μ_3_-PMes). Recrystallization from di­chloro­methane and hexane gave the title compound as yellow platelets.

## Refinement   

Crystal data, data collection and structure refinement details are summarized in Table 1 ▸. Two H atoms bridging Ru atoms were found in a difference-Fourier map and were refined freely. All other H atoms were placed at their geometrically calculated positions with C—H = 0.95 or 0.98 Å. Fifty outliers were omitted in the final refinement.

## Supplementary Material

Crystal structure: contains datablock(s) I. DOI: 10.1107/S2056989017007770/is5476sup1.cif

Structure factors: contains datablock(s) I. DOI: 10.1107/S2056989017007770/is5476Isup2.hkl

CCDC reference: 1552227

Additional supporting information:  crystallographic information; 3D view; checkCIF report

## Figures and Tables

**Table 1:** Experimental details

Crystal data	
Chemical formula	[Ru_3_(C_30_H_24_P_2_)(C_9_H_11_P)(CO)_7_H_2_]
*M*_r_	1097.88
Crystal system, space group	Triclinic, *P* 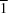
Temperature (K)	150
*a*, *b*, *c* (Å)	10.9732 (2), 19.4127 (3), 22.1186 (2)
α, β, γ (°)	114.1719 (11), 90.2641 (14), 92.5256 (13)
*V* (Å^3^)	4293.07 (11)
*Z*	4
Radiation type	Mo *K*α
μ (mm^−1^)	1.21
Crystal size (mm)	0.20 × 0.20 × 0.10

Data collection	
Diffractometer	Rigaku R-AXIS RAPID imaging plate
Absorption correction	Numerical (*NUMABS*; Higashi, 1999 ▸)
*T*_min_, *T*_max_	0.795, 0.889
No. of measured, independent and observed [*I* > 2σ(*I*)] reflections	30808, 14332, 12888
*R*_int_	0.060
(sin θ/λ)_max_ (Å^−1^)	0.595

Refinement	
*R*[*F* ^2^ > 2σ(*F* ^2^)], *wR*(*F* ^2^), *S*	0.057, 0.146, 1.15
No. of reflections	14332
No. of parameters	1084
H-atom treatment	H atoms treated by a mixture of independent and constrained refinement
Δρ_max_, Δρ_min_ (e Å^−3^)	2.40, −1.06

Computer programs: *PROCESS-AUTO* (Rigaku, 1998 ▸), *TEXSAN* (Molecular Structure Corporation & Rigaku, 2000 ▸), *SHELXS97* and *SHELXL97* (Sheldrick, 2008 ▸) and *Mercury* (Macrae *et al.*, 2008 ▸).

## supplementary crystallographic information

### Crystal data

**Table:**

[Ru_3_(C_30_H_24_P_2_)(C_9_H_11_P)(CO)_7_H_2_]	*Z* = 4
*M**_r_* = 1097.88	*F*(000) = 2184
Triclinic, *P*1	*D*_x_ = 1.699 Mg m^−^^3^
Hall symbol: -P 1	Mo *K*α radiation, λ = 0.71069 Å
*a* = 10.9732 (2) Å	Cell parameters from 17326 reflections
*b* = 19.4127 (3) Å	θ = 3.7–54.9°
*c* = 22.1186 (2) Å	µ = 1.21 mm^−^^1^
α = 114.1719 (11)°	*T* = 150 K
β = 90.2641 (14)°	Platelet, yellow
γ = 92.5256 (13)°	0.20 × 0.20 × 0.10 mm
*V* = 4293.07 (11) Å^3^	

### Data collection

**Table:**

Rigaku R-AXIS RAPID imaging plate diffractometer	14332 independent reflections
Radiation source: fine-focus sealed tube	12888 reflections with *I* > 2σ(*I*)
Graphite monochromator	*R*_int_ = 0.060
ω scans	θ_max_ = 25.0°, θ_min_ = 1.2°
Absorption correction: numerical (NUMABS; Higashi, 1999)	*h* = −13→13
*T*_min_ = 0.795, *T*_max_ = 0.889	*k* = −23→23
30808 measured reflections	*l* = −25→26

### Refinement

**Table:**

Refinement on *F*^2^	Primary atom site location: structure-invariant direct methods
Least-squares matrix: full	Secondary atom site location: difference Fourier map
*R*[*F*^2^ > 2σ(*F*^2^)] = 0.057	Hydrogen site location: inferred from neighbouring sites
*wR*(*F*^2^) = 0.146	H atoms treated by a mixture of independent and constrained refinement
*S* = 1.15	*w* = 1/[σ^2^(*F*_o_^2^) + (0.0424*P*)^2^ + 58.9367*P*] where *P* = (*F*_o_^2^ + 2*F*_c_^2^)/3
14332 reflections	(Δ/σ)_max_ = 0.002
1084 parameters	Δρ_max_ = 2.40 e Å^−^^3^
0 restraints	Δρ_min_ = −1.06 e Å^−^^3^

### Special details

**Table:**

Geometry. All esds (except the esd in the dihedral angle between two l.s. planes) are estimated using the full covariance matrix. The cell esds are taken into account individually in the estimation of esds in distances, angles and torsion angles; correlations between esds in cell parameters are only used when they are defined by crystal symmetry. An approximate (isotropic) treatment of cell esds is used for estimating esds involving l.s. planes.
Refinement. Refinement of F^2^ against ALL reflections. The weighted R-factor wR and goodness of fit S are based on F^2^, conventional R-factors R are based on F, with F set to zero for negative F^2^. The threshold expression of F^2^ > 2sigma(F^2^) is used only for calculating R-factors(gt) etc. and is not relevant to the choice of reflections for refinement. R-factors based on F^2^ are statistically about twice as large as those based on F, and R- factors based on ALL data will be even larger.

### Fractional atomic coordinates and isotropic or equivalent isotropic displacement parameters (Å^2^)

**Table:**

	*x*	*y*	*z*	*U*_iso_*/*U*_eq_	
Ru1	0.31256 (5)	0.26028 (3)	0.75171 (3)	0.01095 (13)	
Ru2	0.46980 (5)	0.14462 (3)	0.66411 (3)	0.01404 (14)	
H1	0.476 (7)	0.245 (4)	0.734 (4)	0.009 (18)*	
Ru3	0.32957 (5)	0.10606 (3)	0.75184 (3)	0.01345 (14)	
H2	0.339 (9)	0.200 (5)	0.795 (5)	0.04 (3)*	
P1	0.25754 (17)	0.14136 (10)	0.67012 (9)	0.0129 (4)	
P2	0.13347 (16)	0.31589 (10)	0.79671 (9)	0.0120 (4)	
P3	0.40423 (17)	0.35848 (10)	0.84357 (9)	0.0129 (4)	
O1	0.3105 (5)	0.3441 (3)	0.6639 (3)	0.0265 (13)	
O2	0.7107 (5)	0.1556 (4)	0.7402 (3)	0.0351 (15)	
O3	0.4961 (6)	−0.0170 (3)	0.5614 (3)	0.0312 (14)	
O4	0.5533 (6)	0.2249 (4)	0.5763 (3)	0.0423 (17)	
O5	0.5549 (6)	0.1033 (3)	0.8335 (3)	0.0290 (13)	
O6	0.3259 (6)	−0.0617 (3)	0.6626 (3)	0.0311 (14)	
O7	0.1338 (6)	0.0692 (4)	0.8334 (3)	0.0357 (15)	
C1	0.3093 (7)	0.3127 (4)	0.6981 (4)	0.0174 (16)	
C2	0.6206 (7)	0.1514 (5)	0.7123 (4)	0.0200 (16)	
C3	0.4858 (7)	0.0438 (5)	0.5995 (4)	0.0213 (17)	
C4	0.5209 (7)	0.1923 (5)	0.6072 (4)	0.0254 (18)	
C5	0.4721 (7)	0.1063 (4)	0.8044 (4)	0.0183 (16)	
C6	0.3291 (7)	0.0008 (4)	0.6957 (4)	0.0165 (15)	
C7	0.2031 (8)	0.0875 (4)	0.8042 (4)	0.0211 (17)	
C8	0.1249 (7)	0.1149 (4)	0.6131 (4)	0.0179 (16)	
C9	0.0131 (7)	0.0926 (4)	0.6318 (4)	0.0170 (15)	
C10	−0.0904 (7)	0.0792 (4)	0.5911 (4)	0.0237 (17)	
H3	−0.1658	0.0648	0.6046	0.028*	
C11	−0.0857 (7)	0.0864 (4)	0.5310 (4)	0.0230 (18)	
C12	0.0259 (8)	0.1060 (4)	0.5117 (4)	0.0231 (18)	
H4	0.0302	0.1102	0.4704	0.028*	
C13	0.1334 (7)	0.1200 (4)	0.5511 (4)	0.0178 (16)	
C14	−0.0027 (7)	0.0839 (5)	0.6958 (4)	0.0229 (17)	
H5	−0.0898	0.0770	0.7027	0.034*	
H6	0.0315	0.1293	0.7326	0.034*	
H7	0.0398	0.0397	0.6938	0.034*	
C15	−0.1997 (8)	0.0746 (5)	0.4889 (5)	0.034 (2)	
H8	−0.1779	0.0753	0.4462	0.051*	
H9	−0.2549	0.1151	0.5116	0.051*	
H10	−0.2403	0.0257	0.4814	0.051*	
C16	0.2493 (8)	0.1401 (5)	0.5258 (4)	0.0268 (18)	
H11	0.2773	0.1919	0.5548	0.040*	
H12	0.2352	0.1363	0.4808	0.040*	
H13	0.3115	0.1051	0.5253	0.040*	
C17	0.0234 (7)	0.3264 (4)	0.7393 (4)	0.0154 (15)	
C18	−0.0664 (7)	0.2694 (4)	0.7084 (4)	0.0187 (16)	
H14	−0.0735	0.2269	0.7193	0.022*	
C19	−0.1455 (7)	0.2749 (5)	0.6616 (4)	0.0224 (17)	
H15	−0.2072	0.2362	0.6410	0.027*	
C20	−0.1357 (7)	0.3356 (5)	0.6447 (4)	0.0235 (17)	
H16	−0.1896	0.3384	0.6122	0.028*	
C21	−0.0471 (8)	0.3926 (5)	0.6752 (4)	0.0247 (18)	
H17	−0.0414	0.4352	0.6644	0.030*	
C22	0.0335 (7)	0.3875 (4)	0.7216 (4)	0.0206 (16)	
H18	0.0958	0.4260	0.7415	0.025*	
C23	0.0430 (7)	0.2847 (4)	0.8510 (3)	0.0140 (14)	
C24	−0.0835 (7)	0.2969 (4)	0.8594 (4)	0.0194 (16)	
H19	−0.1259	0.3175	0.8336	0.023*	
C25	−0.1440 (8)	0.2781 (5)	0.9057 (4)	0.0252 (18)	
H20	−0.2291	0.2850	0.9108	0.030*	
C26	−0.0844 (8)	0.2497 (5)	0.9446 (4)	0.0233 (17)	
H21	−0.1278	0.2375	0.9763	0.028*	
C27	0.0399 (8)	0.2389 (5)	0.9373 (4)	0.0239 (17)	
H22	0.0823	0.2199	0.9643	0.029*	
C28	0.1020 (7)	0.2563 (4)	0.8899 (4)	0.0216 (17)	
H23	0.1868	0.2483	0.8846	0.026*	
C29	0.1736 (7)	0.4141 (4)	0.8553 (4)	0.0148 (15)	
C30	0.0865 (7)	0.4675 (5)	0.8825 (4)	0.0246 (18)	
H24	0.0035	0.4544	0.8685	0.029*	
C31	0.1184 (8)	0.5396 (5)	0.9297 (4)	0.0259 (18)	
H25	0.0577	0.5750	0.9491	0.031*	
C32	0.2408 (8)	0.5591 (4)	0.9481 (4)	0.0219 (17)	
H26	0.2643	0.6089	0.9788	0.026*	
C33	0.3288 (7)	0.5064 (4)	0.9219 (4)	0.0190 (16)	
H27	0.4115	0.5199	0.9363	0.023*	
C34	0.2975 (7)	0.4340 (4)	0.8747 (3)	0.0137 (14)	
C35	0.5467 (7)	0.4072 (4)	0.8360 (4)	0.0159 (15)	
C36	0.5693 (7)	0.4159 (5)	0.7780 (4)	0.0253 (18)	
H28	0.5123	0.3953	0.7418	0.030*	
C37	0.6753 (8)	0.4548 (5)	0.7723 (5)	0.0289 (19)	
H29	0.6895	0.4611	0.7325	0.035*	
C38	0.7601 (8)	0.4843 (5)	0.8242 (5)	0.0282 (19)	
H30	0.8323	0.5108	0.8202	0.034*	
C39	0.7388 (8)	0.4748 (5)	0.8811 (4)	0.0280 (19)	
H31	0.7980	0.4939	0.9163	0.034*	
C40	0.6333 (7)	0.4380 (4)	0.8888 (4)	0.0207 (16)	
H32	0.6191	0.4335	0.9294	0.025*	
C41	0.4316 (7)	0.3373 (4)	0.9156 (3)	0.0161 (15)	
C42	0.5178 (7)	0.2834 (4)	0.9100 (4)	0.0211 (17)	
H33	0.5643	0.2626	0.8710	0.025*	
C43	0.5347 (7)	0.2609 (4)	0.9606 (4)	0.0231 (17)	
H34	0.5942	0.2253	0.9568	0.028*	
C44	0.4660 (8)	0.2894 (4)	1.0168 (4)	0.0246 (18)	
H35	0.4766	0.2722	1.0509	0.029*	
C45	0.3815 (8)	0.3431 (5)	1.0237 (4)	0.0245 (18)	
H36	0.3348	0.3631	1.0626	0.029*	
C46	0.3655 (7)	0.3674 (5)	0.9726 (4)	0.0225 (17)	
H37	0.3089	0.4048	0.9774	0.027*	
Ru4	0.77029 (5)	−0.25228 (3)	0.74916 (3)	0.01190 (13)	
Ru5	0.94605 (5)	−0.13718 (3)	0.83747 (3)	0.01403 (14)	
H38	0.927 (9)	−0.2271	0.769 (5)	0.04 (3)*	
Ru6	0.81076 (5)	−0.09780 (3)	0.75031 (3)	0.01341 (14)	
H39	0.790 (7)	−0.197 (4)	0.707 (4)	0.01 (2)*	
P4	0.73332 (17)	−0.13435 (10)	0.83138 (9)	0.0132 (4)	
P5	0.58284 (17)	−0.30934 (10)	0.70325 (9)	0.0146 (4)	
P6	0.84595 (17)	−0.34968 (10)	0.65773 (9)	0.0143 (4)	
O8	0.7578 (6)	−0.3344 (3)	0.8385 (3)	0.0281 (13)	
O9	1.1827 (6)	−0.1484 (4)	0.7605 (3)	0.0379 (16)	
O10	1.0006 (6)	0.0239 (3)	0.9413 (3)	0.0299 (14)	
O11	1.0190 (6)	−0.2175 (4)	0.9243 (3)	0.0369 (16)	
O12	1.0304 (6)	−0.1009 (3)	0.6637 (3)	0.0315 (14)	
O13	0.8434 (6)	0.0700 (3)	0.8399 (3)	0.0292 (13)	
O14	0.6197 (6)	−0.0579 (4)	0.6711 (3)	0.0366 (16)	
C47	0.7611 (7)	−0.3053 (4)	0.8032 (4)	0.0185 (16)	
C48	1.0960 (7)	−0.1437 (5)	0.7896 (4)	0.0223 (17)	
C49	0.9797 (7)	−0.0358 (5)	0.9024 (4)	0.0194 (16)	
C50	0.9908 (7)	−0.1858 (5)	0.8935 (4)	0.0199 (17)	
C51	0.9495 (7)	−0.1000 (4)	0.6951 (4)	0.0185 (16)	
C52	0.8327 (7)	0.0072 (5)	0.8064 (4)	0.0216 (17)	
C53	0.6867 (8)	−0.0769 (4)	0.6997 (4)	0.0219 (17)	
C54	0.6061 (7)	−0.1109 (4)	0.8879 (3)	0.0135 (14)	
C55	0.4958 (7)	−0.0884 (4)	0.8698 (4)	0.0200 (16)	
C56	0.3966 (8)	−0.0769 (4)	0.9104 (4)	0.0243 (18)	
H40	0.3231	−0.0620	0.8975	0.029*	
C57	0.4001 (8)	−0.0863 (4)	0.9694 (4)	0.0236 (17)	
C58	0.5077 (7)	−0.1054 (4)	0.9881 (4)	0.0196 (16)	
H41	0.5123	−0.1103	1.0291	0.024*	
C59	0.6120 (8)	−0.1182 (4)	0.9492 (4)	0.0227 (17)	
C60	0.4810 (7)	−0.0774 (5)	0.8062 (4)	0.0226 (17)	
H42	0.3980	−0.0623	0.8028	0.034*	
H43	0.4955	−0.1248	0.7681	0.034*	
H44	0.5400	−0.0378	0.8065	0.034*	
C61	0.2873 (8)	−0.0769 (5)	1.0112 (4)	0.0282 (19)	
H45	0.2374	−0.1243	0.9943	0.042*	
H46	0.2397	−0.0365	1.0087	0.042*	
H47	0.3121	−0.0637	1.0573	0.042*	
C62	0.7273 (8)	−0.1390 (5)	0.9740 (4)	0.0247 (18)	
H48	0.7129	−0.1404	1.0172	0.037*	
H49	0.7930	−0.1012	0.9786	0.037*	
H50	0.7507	−0.1888	0.9424	0.037*	
C63	0.4730 (6)	−0.3206 (4)	0.7608 (4)	0.0168 (15)	
C64	0.3901 (7)	−0.2650 (4)	0.7910 (4)	0.0218 (17)	
H51	0.3859	−0.2236	0.7788	0.026*	
C65	0.3129 (8)	−0.2703 (5)	0.8395 (4)	0.0275 (19)	
H52	0.2565	−0.2323	0.8602	0.033*	
C66	0.3185 (8)	−0.3300 (5)	0.8571 (4)	0.0276 (19)	
H53	0.2656	−0.3334	0.8897	0.033*	
C67	0.4010 (8)	−0.3855 (5)	0.8274 (4)	0.0275 (19)	
H54	0.4041	−0.4271	0.8394	0.033*	
C68	0.4787 (7)	−0.3804 (5)	0.7803 (4)	0.0237 (17)	
H55	0.5366	−0.4179	0.7610	0.028*	
C69	0.4935 (7)	−0.2793 (4)	0.6491 (4)	0.0198 (16)	
C70	0.3682 (7)	−0.2961 (5)	0.6383 (4)	0.0234 (17)	
H56	0.3255	−0.3215	0.6611	0.028*	
C71	0.3065 (8)	−0.2754 (5)	0.5940 (4)	0.0282 (19)	
H57	0.2209	−0.2855	0.5875	0.034*	
C72	0.3678 (8)	−0.2403 (5)	0.5592 (4)	0.0256 (18)	
H58	0.3242	−0.2269	0.5287	0.031*	
C73	0.4930 (8)	−0.2245 (5)	0.5684 (4)	0.0246 (18)	
H59	0.5360	−0.2007	0.5444	0.029*	
C74	0.5532 (8)	−0.2443 (4)	0.6137 (4)	0.0232 (17)	
H60	0.6386	−0.2334	0.6206	0.028*	
C75	0.6098 (7)	−0.4080 (4)	0.6468 (4)	0.0181 (15)	
C76	0.5137 (8)	−0.4625 (5)	0.6205 (4)	0.0277 (19)	
H61	0.4317	−0.4492	0.6311	0.033*	
C77	0.5391 (9)	−0.5362 (5)	0.5787 (5)	0.036 (2)	
H62	0.4743	−0.5736	0.5614	0.043*	
C78	0.6579 (8)	−0.5555 (5)	0.5621 (4)	0.030 (2)	
H63	0.6740	−0.6061	0.5336	0.036*	
C79	0.7532 (8)	−0.5022 (5)	0.5864 (4)	0.0256 (18)	
H64	0.8345	−0.5162	0.5748	0.031*	
C80	0.7305 (7)	−0.4267 (4)	0.6286 (4)	0.0177 (15)	
C81	0.9866 (6)	−0.3947 (4)	0.6631 (4)	0.0159 (15)	
C82	1.0196 (7)	−0.3937 (5)	0.7252 (4)	0.0230 (17)	
H65	0.9723	−0.3678	0.7629	0.028*	
C83	1.1215 (8)	−0.4307 (5)	0.7308 (5)	0.0292 (19)	
H66	1.1440	−0.4300	0.7726	0.035*	
C84	1.1894 (8)	−0.4680 (5)	0.6770 (5)	0.031 (2)	
H67	1.2583	−0.4938	0.6814	0.037*	
C85	1.1591 (8)	−0.4688 (5)	0.6156 (5)	0.032 (2)	
H68	1.2076	−0.4943	0.5783	0.038*	
C86	1.0577 (8)	−0.4322 (5)	0.6092 (4)	0.0268 (18)	
H69	1.0367	−0.4328	0.5672	0.032*	
C87	0.8699 (7)	−0.3309 (4)	0.5843 (4)	0.0189 (16)	
C88	0.9687 (8)	−0.2832 (5)	0.5841 (4)	0.0252 (18)	
H70	1.0245	−0.2641	0.6209	0.030*	
C89	0.9870 (8)	−0.2634 (5)	0.5320 (4)	0.0266 (18)	
H71	1.0546	−0.2307	0.5328	0.032*	
C90	0.9049 (9)	−0.2917 (5)	0.4772 (4)	0.033 (2)	
H72	0.9176	−0.2790	0.4405	0.039*	
C91	0.8061 (9)	−0.3379 (5)	0.4773 (4)	0.033 (2)	
H73	0.7495	−0.3560	0.4409	0.039*	
C92	0.7887 (9)	−0.3582 (5)	0.5301 (4)	0.0275 (19)	
H74	0.7210	−0.3908	0.5293	0.033*	

### Atomic displacement parameters (Å^2^)

**Table:**

	*U*^11^	*U*^22^	*U*^33^	*U*^12^	*U*^13^	*U*^23^
Ru1	0.0100 (3)	0.0136 (3)	0.0100 (3)	0.0008 (2)	0.0009 (2)	0.0056 (2)
Ru2	0.0106 (3)	0.0204 (3)	0.0123 (3)	0.0038 (2)	0.0026 (2)	0.0075 (2)
Ru3	0.0132 (3)	0.0146 (3)	0.0143 (3)	0.0020 (2)	0.0007 (2)	0.0077 (2)
P1	0.0149 (9)	0.0120 (8)	0.0115 (8)	0.0022 (7)	−0.0023 (7)	0.0045 (7)
P2	0.0106 (9)	0.0128 (8)	0.0127 (8)	0.0010 (7)	0.0004 (7)	0.0053 (7)
P3	0.0115 (9)	0.0160 (9)	0.0119 (8)	−0.0004 (7)	−0.0006 (7)	0.0066 (7)
O1	0.030 (3)	0.034 (3)	0.025 (3)	0.000 (3)	−0.001 (3)	0.022 (3)
O2	0.015 (3)	0.062 (4)	0.033 (3)	0.006 (3)	−0.001 (3)	0.025 (3)
O3	0.035 (4)	0.033 (4)	0.023 (3)	0.015 (3)	0.007 (3)	0.007 (3)
O4	0.036 (4)	0.070 (5)	0.037 (4)	−0.006 (3)	0.004 (3)	0.038 (4)
O5	0.030 (3)	0.030 (3)	0.028 (3)	0.003 (3)	−0.009 (3)	0.013 (3)
O6	0.036 (4)	0.018 (3)	0.035 (3)	0.004 (2)	0.001 (3)	0.007 (3)
O7	0.035 (4)	0.044 (4)	0.039 (4)	0.005 (3)	0.017 (3)	0.028 (3)
C1	0.019 (4)	0.021 (4)	0.013 (3)	0.005 (3)	−0.001 (3)	0.007 (3)
C2	0.017 (4)	0.029 (4)	0.021 (4)	0.003 (3)	0.014 (3)	0.016 (3)
C3	0.018 (4)	0.031 (5)	0.017 (4)	0.008 (3)	0.004 (3)	0.011 (4)
C4	0.013 (4)	0.039 (5)	0.023 (4)	0.003 (3)	0.000 (3)	0.010 (4)
C5	0.018 (4)	0.020 (4)	0.017 (4)	0.006 (3)	0.013 (3)	0.008 (3)
C6	0.016 (4)	0.021 (4)	0.016 (4)	0.004 (3)	0.000 (3)	0.010 (3)
C7	0.029 (4)	0.018 (4)	0.017 (4)	0.001 (3)	0.004 (3)	0.008 (3)
C8	0.018 (4)	0.016 (4)	0.017 (4)	0.006 (3)	−0.007 (3)	0.004 (3)
C9	0.018 (4)	0.011 (3)	0.021 (4)	−0.001 (3)	0.002 (3)	0.006 (3)
C10	0.015 (4)	0.023 (4)	0.026 (4)	0.001 (3)	−0.002 (3)	0.004 (3)
C11	0.024 (4)	0.011 (3)	0.025 (4)	0.008 (3)	−0.009 (3)	−0.002 (3)
C12	0.027 (5)	0.023 (4)	0.018 (4)	0.009 (3)	−0.005 (3)	0.006 (3)
C13	0.022 (4)	0.014 (4)	0.016 (4)	0.004 (3)	−0.002 (3)	0.003 (3)
C14	0.013 (4)	0.029 (4)	0.028 (4)	−0.001 (3)	0.000 (3)	0.014 (4)
C15	0.023 (5)	0.029 (5)	0.043 (5)	0.003 (4)	−0.014 (4)	0.007 (4)
C16	0.027 (5)	0.036 (5)	0.021 (4)	0.003 (4)	−0.003 (4)	0.015 (4)
C17	0.015 (4)	0.019 (4)	0.016 (3)	0.010 (3)	0.003 (3)	0.010 (3)
C18	0.019 (4)	0.016 (4)	0.022 (4)	0.002 (3)	−0.005 (3)	0.008 (3)
C19	0.017 (4)	0.026 (4)	0.024 (4)	0.005 (3)	0.001 (3)	0.010 (3)
C20	0.018 (4)	0.032 (4)	0.021 (4)	0.004 (3)	−0.005 (3)	0.010 (3)
C21	0.029 (5)	0.034 (5)	0.024 (4)	0.009 (4)	0.003 (4)	0.024 (4)
C22	0.012 (4)	0.024 (4)	0.028 (4)	−0.002 (3)	−0.001 (3)	0.012 (3)
C23	0.014 (4)	0.014 (3)	0.013 (3)	−0.001 (3)	0.003 (3)	0.005 (3)
C24	0.017 (4)	0.026 (4)	0.016 (4)	0.006 (3)	0.002 (3)	0.009 (3)
C25	0.020 (4)	0.033 (5)	0.020 (4)	0.003 (3)	0.006 (3)	0.007 (4)
C26	0.027 (4)	0.029 (4)	0.017 (4)	−0.003 (3)	0.008 (3)	0.013 (3)
C27	0.026 (4)	0.030 (4)	0.020 (4)	0.007 (3)	0.004 (3)	0.015 (4)
C28	0.019 (4)	0.026 (4)	0.020 (4)	0.008 (3)	0.005 (3)	0.010 (3)
C29	0.016 (4)	0.015 (3)	0.013 (3)	−0.001 (3)	−0.003 (3)	0.006 (3)
C30	0.017 (4)	0.027 (4)	0.028 (4)	0.002 (3)	−0.006 (3)	0.009 (4)
C31	0.028 (5)	0.018 (4)	0.029 (4)	0.003 (3)	0.002 (4)	0.007 (3)
C32	0.029 (4)	0.014 (4)	0.020 (4)	0.000 (3)	0.003 (3)	0.005 (3)
C33	0.019 (4)	0.017 (4)	0.021 (4)	−0.001 (3)	−0.005 (3)	0.008 (3)
C34	0.014 (4)	0.018 (4)	0.011 (3)	0.002 (3)	0.003 (3)	0.008 (3)
C35	0.015 (4)	0.007 (3)	0.022 (4)	0.002 (3)	−0.003 (3)	0.003 (3)
C36	0.018 (4)	0.034 (5)	0.025 (4)	−0.006 (3)	−0.001 (3)	0.013 (4)
C37	0.020 (4)	0.042 (5)	0.040 (5)	−0.004 (4)	0.004 (4)	0.033 (4)
C38	0.024 (4)	0.021 (4)	0.041 (5)	−0.002 (3)	0.010 (4)	0.014 (4)
C39	0.020 (4)	0.024 (4)	0.032 (5)	−0.004 (3)	−0.009 (4)	0.003 (4)
C40	0.022 (4)	0.017 (4)	0.021 (4)	−0.003 (3)	−0.003 (3)	0.006 (3)
C41	0.015 (4)	0.021 (4)	0.011 (3)	−0.003 (3)	−0.005 (3)	0.006 (3)
C42	0.019 (4)	0.021 (4)	0.024 (4)	−0.001 (3)	−0.008 (3)	0.010 (3)
C43	0.020 (4)	0.021 (4)	0.031 (4)	−0.004 (3)	−0.011 (4)	0.014 (4)
C44	0.038 (5)	0.023 (4)	0.016 (4)	−0.010 (3)	−0.006 (4)	0.013 (3)
C45	0.031 (5)	0.028 (4)	0.013 (4)	0.003 (3)	0.004 (3)	0.007 (3)
C46	0.024 (4)	0.027 (4)	0.020 (4)	0.005 (3)	−0.002 (3)	0.013 (3)
Ru4	0.0126 (3)	0.0127 (3)	0.0111 (3)	0.0000 (2)	0.0016 (2)	0.0058 (2)
Ru5	0.0115 (3)	0.0191 (3)	0.0125 (3)	−0.0007 (2)	0.0012 (2)	0.0077 (2)
Ru6	0.0132 (3)	0.0149 (3)	0.0143 (3)	−0.0004 (2)	0.0016 (2)	0.0083 (2)
P4	0.0135 (9)	0.0140 (9)	0.0118 (8)	−0.0007 (7)	0.0037 (7)	0.0050 (7)
P5	0.0135 (9)	0.0163 (9)	0.0144 (9)	−0.0009 (7)	0.0008 (7)	0.0070 (7)
P6	0.0138 (9)	0.0161 (9)	0.0133 (9)	0.0006 (7)	0.0028 (7)	0.0063 (7)
O8	0.037 (4)	0.031 (3)	0.027 (3)	−0.006 (3)	−0.003 (3)	0.024 (3)
O9	0.018 (3)	0.066 (5)	0.041 (4)	0.002 (3)	0.013 (3)	0.033 (4)
O10	0.031 (3)	0.025 (3)	0.024 (3)	−0.007 (3)	−0.004 (3)	0.001 (3)
O11	0.030 (4)	0.058 (4)	0.041 (4)	0.008 (3)	0.000 (3)	0.038 (4)
O12	0.037 (4)	0.028 (3)	0.032 (3)	0.004 (3)	0.021 (3)	0.015 (3)
O13	0.034 (3)	0.017 (3)	0.032 (3)	0.002 (2)	0.001 (3)	0.006 (3)
O14	0.037 (4)	0.040 (4)	0.045 (4)	0.003 (3)	−0.012 (3)	0.030 (3)
C47	0.016 (4)	0.019 (4)	0.020 (4)	−0.005 (3)	0.001 (3)	0.009 (3)
C48	0.014 (4)	0.029 (4)	0.026 (4)	0.004 (3)	−0.002 (4)	0.013 (4)
C49	0.011 (4)	0.030 (5)	0.021 (4)	−0.006 (3)	0.002 (3)	0.016 (4)
C50	0.008 (4)	0.037 (5)	0.012 (3)	0.000 (3)	0.006 (3)	0.008 (3)
C51	0.025 (4)	0.014 (4)	0.019 (4)	−0.002 (3)	0.007 (4)	0.010 (3)
C52	0.016 (4)	0.032 (5)	0.022 (4)	0.000 (3)	−0.001 (3)	0.016 (4)
C53	0.029 (4)	0.015 (4)	0.023 (4)	0.002 (3)	0.001 (4)	0.008 (3)
C54	0.015 (4)	0.012 (3)	0.013 (3)	0.000 (3)	0.006 (3)	0.006 (3)
C55	0.022 (4)	0.018 (4)	0.022 (4)	0.002 (3)	0.001 (3)	0.010 (3)
C56	0.022 (4)	0.017 (4)	0.033 (5)	0.001 (3)	0.006 (4)	0.009 (3)
C57	0.026 (4)	0.015 (4)	0.026 (4)	0.001 (3)	0.015 (4)	0.004 (3)
C58	0.024 (4)	0.021 (4)	0.014 (4)	−0.005 (3)	0.012 (3)	0.008 (3)
C59	0.028 (5)	0.019 (4)	0.020 (4)	−0.002 (3)	0.000 (3)	0.007 (3)
C60	0.015 (4)	0.028 (4)	0.030 (4)	0.001 (3)	0.005 (3)	0.017 (4)
C61	0.024 (4)	0.030 (5)	0.023 (4)	0.004 (3)	0.016 (4)	0.002 (4)
C62	0.033 (5)	0.028 (4)	0.015 (4)	0.002 (4)	0.007 (3)	0.011 (3)
C63	0.010 (4)	0.022 (4)	0.018 (4)	−0.005 (3)	0.001 (3)	0.009 (3)
C64	0.025 (4)	0.023 (4)	0.021 (4)	−0.002 (3)	−0.003 (3)	0.013 (3)
C65	0.025 (5)	0.027 (4)	0.020 (4)	−0.007 (3)	0.011 (4)	0.000 (3)
C66	0.021 (4)	0.041 (5)	0.019 (4)	−0.013 (4)	0.001 (3)	0.012 (4)
C67	0.031 (5)	0.033 (5)	0.028 (4)	−0.006 (4)	−0.003 (4)	0.023 (4)
C68	0.016 (4)	0.031 (4)	0.028 (4)	−0.001 (3)	0.003 (3)	0.015 (4)
C69	0.023 (4)	0.023 (4)	0.016 (4)	0.000 (3)	0.001 (3)	0.010 (3)
C70	0.023 (4)	0.026 (4)	0.019 (4)	−0.002 (3)	−0.004 (3)	0.006 (3)
C71	0.016 (4)	0.045 (5)	0.029 (4)	0.002 (4)	−0.003 (4)	0.021 (4)
C72	0.027 (5)	0.028 (4)	0.024 (4)	0.008 (3)	−0.004 (4)	0.013 (4)
C73	0.031 (5)	0.031 (4)	0.015 (4)	−0.007 (4)	−0.001 (3)	0.014 (3)
C74	0.024 (4)	0.024 (4)	0.023 (4)	−0.005 (3)	−0.006 (3)	0.011 (3)
C75	0.016 (4)	0.020 (4)	0.022 (4)	−0.001 (3)	0.001 (3)	0.012 (3)
C76	0.023 (4)	0.023 (4)	0.032 (5)	−0.005 (3)	0.000 (4)	0.007 (4)
C77	0.032 (5)	0.025 (5)	0.045 (6)	−0.005 (4)	−0.004 (4)	0.008 (4)
C78	0.035 (5)	0.013 (4)	0.031 (5)	−0.004 (3)	0.003 (4)	−0.001 (3)
C79	0.022 (4)	0.026 (4)	0.025 (4)	0.004 (3)	−0.001 (3)	0.006 (4)
C80	0.024 (4)	0.017 (4)	0.013 (3)	0.003 (3)	0.002 (3)	0.007 (3)
C81	0.007 (3)	0.019 (4)	0.019 (4)	0.002 (3)	0.006 (3)	0.005 (3)
C82	0.022 (4)	0.025 (4)	0.023 (4)	0.004 (3)	−0.002 (3)	0.011 (3)
C83	0.028 (5)	0.031 (5)	0.036 (5)	−0.002 (4)	−0.006 (4)	0.021 (4)
C84	0.018 (4)	0.024 (4)	0.051 (6)	0.004 (3)	0.002 (4)	0.017 (4)
C85	0.024 (5)	0.022 (4)	0.048 (6)	0.004 (3)	0.008 (4)	0.013 (4)
C86	0.029 (5)	0.026 (4)	0.029 (4)	0.000 (3)	0.007 (4)	0.014 (4)
C87	0.019 (4)	0.016 (4)	0.019 (4)	0.005 (3)	0.009 (3)	0.004 (3)
C88	0.026 (4)	0.027 (4)	0.024 (4)	0.009 (3)	0.005 (4)	0.011 (4)
C89	0.028 (5)	0.028 (4)	0.032 (5)	0.007 (3)	0.018 (4)	0.019 (4)
C90	0.048 (6)	0.036 (5)	0.021 (4)	0.017 (4)	0.014 (4)	0.017 (4)
C91	0.052 (6)	0.029 (5)	0.018 (4)	−0.004 (4)	−0.003 (4)	0.011 (4)
C92	0.038 (5)	0.025 (4)	0.018 (4)	−0.009 (4)	−0.001 (4)	0.008 (3)

### Geometric parameters (Å, º)

**Table:**

Ru1—C1	1.853 (7)	Ru4—C47	1.871 (8)
Ru1—P2	2.3115 (19)	Ru4—P5	2.3126 (19)
Ru1—P1	2.3252 (18)	Ru4—P6	2.3187 (19)
Ru1—P3	2.3267 (19)	Ru4—P4	2.3221 (19)
Ru1—Ru2	2.9297 (8)	Ru4—Ru5	2.9220 (8)
Ru1—Ru3	3.0089 (8)	Ru4—Ru6	3.0018 (8)
Ru1—H1	1.85 (8)	Ru4—H38	1.77 (10)
Ru1—H2	1.82 (10)	Ru4—H39	1.71 (8)
Ru2—C3	1.909 (8)	Ru5—C50	1.914 (8)
Ru2—C4	1.915 (9)	Ru5—C49	1.920 (8)
Ru2—C2	1.935 (8)	Ru5—C48	1.942 (9)
Ru2—P1	2.3332 (19)	Ru5—P4	2.3419 (19)
Ru2—Ru3	2.7945 (8)	Ru5—Ru6	2.7902 (8)
Ru2—H1	1.93 (7)	Ru5—H38	1.78 (5)
Ru3—C6	1.908 (8)	Ru6—C52	1.906 (9)
Ru3—C7	1.926 (8)	Ru6—C53	1.918 (8)
Ru3—C5	1.943 (8)	Ru6—C51	1.948 (8)
Ru3—P1	2.3259 (18)	Ru6—P4	2.3331 (19)
Ru3—H2	1.67 (10)	Ru6—H39	1.77 (8)
P1—C8	1.833 (7)	P4—C54	1.823 (7)
P2—C17	1.826 (7)	P5—C63	1.828 (8)
P2—C23	1.830 (7)	P5—C69	1.829 (8)
P2—C29	1.845 (7)	P5—C75	1.847 (8)
P3—C34	1.824 (7)	P6—C80	1.815 (8)
P3—C41	1.828 (7)	P6—C87	1.821 (8)
P3—C35	1.835 (8)	P6—C81	1.834 (7)
O1—C1	1.150 (9)	O8—C47	1.135 (9)
O2—C2	1.144 (10)	O9—C48	1.136 (10)
O3—C3	1.148 (10)	O10—C49	1.138 (10)
O4—C4	1.155 (11)	O11—C50	1.137 (10)
O5—C5	1.130 (10)	O12—C51	1.125 (10)
O6—C6	1.130 (9)	O13—C52	1.138 (10)
O7—C7	1.136 (10)	O14—C53	1.134 (10)
C8—C9	1.403 (11)	C54—C55	1.415 (10)
C8—C13	1.418 (11)	C54—C59	1.420 (11)
C9—C10	1.394 (11)	C55—C56	1.381 (12)
C9—C14	1.502 (11)	C55—C60	1.517 (11)
C10—C11	1.393 (12)	C56—C57	1.390 (12)
C10—H3	0.9500	C56—H40	0.9500
C11—C12	1.387 (13)	C57—C58	1.366 (12)
C11—C15	1.508 (11)	C57—C61	1.521 (11)
C12—C13	1.411 (11)	C58—C59	1.404 (12)
C12—H4	0.9500	C58—H41	0.9500
C13—C16	1.490 (12)	C59—C62	1.512 (11)
C14—H5	0.9800	C60—H42	0.9800
C14—H6	0.9800	C60—H43	0.9800
C14—H7	0.9800	C60—H44	0.9800
C15—H8	0.9800	C61—H45	0.9800
C15—H9	0.9800	C61—H46	0.9800
C15—H10	0.9800	C61—H47	0.9800
C16—H11	0.9800	C62—H48	0.9800
C16—H12	0.9800	C62—H49	0.9800
C16—H13	0.9800	C62—H50	0.9800
C17—C18	1.393 (11)	C63—C64	1.394 (11)
C17—C22	1.394 (11)	C63—C68	1.398 (11)
C18—C19	1.388 (11)	C64—C65	1.404 (12)
C18—H14	0.9500	C64—H51	0.9500
C19—C20	1.373 (12)	C65—C66	1.369 (13)
C19—H15	0.9500	C65—H52	0.9500
C20—C21	1.383 (12)	C66—C67	1.388 (13)
C20—H16	0.9500	C66—H53	0.9500
C21—C22	1.387 (11)	C67—C68	1.381 (12)
C21—H17	0.9500	C67—H54	0.9500
C22—H18	0.9500	C68—H55	0.9500
C23—C28	1.372 (11)	C69—C74	1.380 (11)
C23—C24	1.418 (10)	C69—C70	1.396 (11)
C24—C25	1.385 (12)	C70—C71	1.387 (11)
C24—H19	0.9500	C70—H56	0.9500
C25—C26	1.373 (12)	C71—C72	1.379 (12)
C25—H20	0.9500	C71—H57	0.9500
C26—C27	1.389 (12)	C72—C73	1.390 (12)
C26—H21	0.9500	C72—H58	0.9500
C27—C28	1.398 (11)	C73—C74	1.386 (11)
C27—H22	0.9500	C73—H59	0.9500
C28—H23	0.9500	C74—H60	0.9500
C29—C30	1.390 (11)	C75—C76	1.401 (11)
C29—C34	1.412 (10)	C75—C80	1.405 (11)
C30—C31	1.388 (12)	C76—C77	1.391 (13)
C30—H24	0.9500	C76—H61	0.9500
C31—C32	1.392 (12)	C77—C78	1.381 (13)
C31—H25	0.9500	C77—H62	0.9500
C32—C33	1.385 (11)	C78—C79	1.378 (12)
C32—H26	0.9500	C78—H63	0.9500
C33—C34	1.392 (10)	C79—C80	1.412 (11)
C33—H27	0.9500	C79—H64	0.9500
C35—C36	1.385 (11)	C81—C86	1.380 (11)
C35—C40	1.411 (10)	C81—C82	1.411 (11)
C36—C37	1.392 (12)	C82—C83	1.389 (11)
C36—H28	0.9500	C82—H65	0.9500
C37—C38	1.384 (13)	C83—C84	1.358 (13)
C37—H29	0.9500	C83—H66	0.9500
C38—C39	1.365 (13)	C84—C85	1.391 (14)
C38—H30	0.9500	C84—H67	0.9500
C39—C40	1.382 (12)	C85—C86	1.384 (12)
C39—H31	0.9500	C85—H68	0.9500
C40—H32	0.9500	C86—H69	0.9500
C41—C46	1.378 (11)	C87—C92	1.394 (11)
C41—C42	1.410 (11)	C87—C88	1.395 (12)
C42—C43	1.373 (11)	C88—C89	1.369 (12)
C42—H33	0.9500	C88—H70	0.9500
C43—C44	1.379 (12)	C89—C90	1.408 (13)
C43—H34	0.9500	C89—H71	0.9500
C44—C45	1.388 (12)	C90—C91	1.375 (14)
C44—H35	0.9500	C90—H72	0.9500
C45—C46	1.404 (11)	C91—C92	1.389 (12)
C45—H36	0.9500	C91—H73	0.9500
C46—H37	0.9500	C92—H74	0.9500
			
C1—Ru1—P2	87.6 (2)	C47—Ru4—P5	87.9 (2)
C1—Ru1—P1	97.1 (2)	C47—Ru4—P6	96.1 (2)
P2—Ru1—P1	106.74 (7)	P5—Ru4—P6	83.67 (7)
C1—Ru1—P3	96.2 (2)	C47—Ru4—P4	96.6 (2)
P2—Ru1—P3	84.03 (7)	P5—Ru4—P4	106.98 (7)
P1—Ru1—P3	163.17 (7)	P6—Ru4—P4	163.70 (7)
C1—Ru1—Ru2	94.3 (2)	C47—Ru4—Ru5	93.6 (2)
P2—Ru1—Ru2	157.89 (5)	P5—Ru4—Ru5	158.48 (5)
P1—Ru1—Ru2	51.15 (5)	P6—Ru4—Ru5	117.43 (5)
P3—Ru1—Ru2	117.56 (5)	P4—Ru4—Ru5	51.51 (5)
C1—Ru1—Ru3	144.2 (2)	C47—Ru4—Ru6	143.8 (2)
P2—Ru1—Ru3	112.36 (5)	P5—Ru4—Ru6	112.95 (5)
P1—Ru1—Ru3	49.70 (5)	P6—Ru4—Ru6	114.79 (5)
P3—Ru1—Ru3	114.56 (5)	P4—Ru4—Ru6	50.01 (5)
Ru2—Ru1—Ru3	56.123 (19)	Ru5—Ru4—Ru6	56.180 (19)
C1—Ru1—H1	89 (2)	C47—Ru4—H38	92 (3)
P2—Ru1—H1	162 (2)	P5—Ru4—H38	166.7 (19)
P1—Ru1—H1	91 (2)	P6—Ru4—H38	83 (2)
P3—Ru1—H1	79 (2)	P4—Ru4—H38	86.2 (16)
Ru2—Ru1—H1	40 (2)	Ru5—Ru4—H38	34.8 (18)
Ru3—Ru1—H1	80 (2)	Ru6—Ru4—H38	74.1 (16)
C1—Ru1—H2	169 (3)	C47—Ru4—H39	174 (3)
P2—Ru1—H2	103 (3)	P5—Ru4—H39	98 (3)
P1—Ru1—H2	78 (3)	P6—Ru4—H39	87 (3)
P3—Ru1—H2	87 (3)	P4—Ru4—H39	79 (3)
Ru2—Ru1—H2	75 (3)	Ru5—Ru4—H39	80 (3)
Ru3—Ru1—H2	29 (3)	Ru6—Ru4—H39	31 (3)
H1—Ru1—H2	82 (4)	H38—Ru4—H39	83 (4)
C3—Ru2—C4	95.3 (4)	C50—Ru5—C49	95.8 (3)
C3—Ru2—C2	96.4 (3)	C50—Ru5—C48	100.4 (3)
C4—Ru2—C2	100.8 (3)	C49—Ru5—C48	95.9 (3)
C3—Ru2—P1	98.0 (2)	C50—Ru5—P4	109.9 (2)
C4—Ru2—P1	110.0 (2)	C49—Ru5—P4	98.4 (2)
C2—Ru2—P1	144.4 (2)	C48—Ru5—P4	144.8 (2)
C3—Ru2—Ru3	96.4 (2)	C50—Ru5—Ru6	160.7 (2)
C4—Ru2—Ru3	160.7 (2)	C49—Ru5—Ru6	96.2 (2)
C2—Ru2—Ru3	93.2 (2)	C48—Ru5—Ru6	93.4 (2)
P1—Ru2—Ru3	53.03 (5)	P4—Ru5—Ru6	53.21 (5)
C3—Ru2—Ru1	148.7 (2)	C50—Ru5—Ru4	99.2 (2)
C4—Ru2—Ru1	99.3 (3)	C49—Ru5—Ru4	148.9 (2)
C2—Ru2—Ru1	107.8 (2)	C48—Ru5—Ru4	107.8 (2)
P1—Ru2—Ru1	50.91 (5)	P4—Ru5—Ru4	50.90 (5)
Ru3—Ru2—Ru1	63.37 (2)	Ru6—Ru5—Ru4	63.36 (2)
C3—Ru2—H1	172 (2)	C50—Ru5—H38	90 (3)
C4—Ru2—H1	86 (2)	C49—Ru5—H38	172 (3)
C2—Ru2—H1	76 (2)	C48—Ru5—H38	77 (3)
P1—Ru2—H1	89 (2)	P4—Ru5—H38	85 (3)
Ru3—Ru2—H1	85 (2)	Ru6—Ru5—H38	80 (3)
Ru1—Ru2—H1	38 (2)	Ru4—Ru5—H38	34 (3)
C6—Ru3—C7	90.6 (3)	C52—Ru6—C53	90.5 (3)
C6—Ru3—C5	95.2 (3)	C52—Ru6—C51	95.2 (3)
C7—Ru3—C5	99.7 (3)	C53—Ru6—C51	98.8 (3)
C6—Ru3—P1	95.1 (2)	C52—Ru6—P4	96.2 (2)
C7—Ru3—P1	113.9 (2)	C53—Ru6—P4	113.5 (3)
C5—Ru3—P1	144.7 (2)	C51—Ru6—P4	145.6 (2)
C6—Ru3—Ru2	92.4 (2)	C52—Ru6—Ru5	91.8 (2)
C7—Ru3—Ru2	167.0 (2)	C53—Ru6—Ru5	166.9 (3)
C5—Ru3—Ru2	92.6 (2)	C51—Ru6—Ru5	93.8 (2)
P1—Ru3—Ru2	53.27 (5)	P4—Ru6—Ru5	53.50 (5)
C6—Ru3—Ru1	143.5 (2)	C52—Ru6—Ru4	144.0 (2)
C7—Ru3—Ru1	110.9 (2)	C53—Ru6—Ru4	111.7 (2)
C5—Ru3—Ru1	109.1 (2)	C51—Ru6—Ru4	108.1 (2)
P1—Ru3—Ru1	49.68 (5)	P4—Ru6—Ru4	49.69 (5)
Ru2—Ru3—Ru1	60.50 (2)	Ru5—Ru6—Ru4	60.465 (19)
C6—Ru3—H2	174 (3)	C52—Ru6—H39	174 (3)
C7—Ru3—H2	95 (3)	C53—Ru6—H39	94 (3)
C5—Ru3—H2	85 (3)	C51—Ru6—H39	89 (3)
P1—Ru3—H2	81 (3)	P4—Ru6—H39	78 (3)
Ru2—Ru3—H2	81 (3)	Ru5—Ru6—H39	83 (3)
Ru1—Ru3—H2	32 (3)	Ru4—Ru6—H39	30 (3)
C8—P1—Ru1	127.4 (2)	C54—P4—Ru4	126.8 (2)
C8—P1—Ru3	135.9 (3)	C54—P4—Ru6	137.3 (2)
Ru1—P1—Ru3	80.62 (6)	Ru4—P4—Ru6	80.31 (6)
C8—P1—Ru2	138.0 (3)	C54—P4—Ru5	138.0 (2)
Ru1—P1—Ru2	77.94 (6)	Ru4—P4—Ru5	77.58 (6)
Ru3—P1—Ru2	73.71 (5)	Ru6—P4—Ru5	73.28 (6)
C17—P2—C23	105.4 (3)	C63—P5—C69	104.9 (4)
C17—P2—C29	103.7 (3)	C63—P5—C75	102.8 (3)
C23—P2—C29	99.4 (3)	C69—P5—C75	101.0 (3)
C17—P2—Ru1	116.4 (2)	C63—P5—Ru4	115.9 (2)
C23—P2—Ru1	121.6 (2)	C69—P5—Ru4	122.5 (3)
C29—P2—Ru1	107.6 (2)	C75—P5—Ru4	107.0 (2)
C34—P3—C41	102.6 (3)	C80—P6—C87	102.3 (3)
C34—P3—C35	103.6 (3)	C80—P6—C81	104.3 (3)
C41—P3—C35	104.2 (3)	C87—P6—C81	102.2 (3)
C34—P3—Ru1	107.8 (2)	C80—P6—Ru4	107.7 (3)
C41—P3—Ru1	115.6 (2)	C87—P6—Ru4	116.3 (2)
C35—P3—Ru1	120.9 (2)	C81—P6—Ru4	121.8 (2)
O1—C1—Ru1	177.8 (7)	O8—C47—Ru4	176.6 (7)
O2—C2—Ru2	179.1 (7)	O9—C48—Ru5	178.8 (8)
O3—C3—Ru2	179.1 (7)	O10—C49—Ru5	179.1 (7)
O4—C4—Ru2	175.9 (8)	O11—C50—Ru5	176.8 (7)
O5—C5—Ru3	177.2 (7)	O12—C51—Ru6	179.3 (8)
O6—C6—Ru3	178.4 (7)	O13—C52—Ru6	178.7 (7)
O7—C7—Ru3	172.6 (7)	O14—C53—Ru6	173.2 (7)
C9—C8—C13	119.7 (7)	C55—C54—C59	118.2 (7)
C9—C8—P1	120.0 (6)	C55—C54—P4	120.1 (6)
C13—C8—P1	120.2 (6)	C59—C54—P4	121.6 (6)
C10—C9—C8	120.0 (7)	C56—C55—C54	119.6 (7)
C10—C9—C14	116.9 (7)	C56—C55—C60	118.0 (7)
C8—C9—C14	123.0 (7)	C54—C55—C60	122.3 (7)
C11—C10—C9	121.5 (8)	C55—C56—C57	122.6 (8)
C11—C10—H3	119.3	C55—C56—H40	118.7
C9—C10—H3	119.3	C57—C56—H40	118.7
C12—C11—C10	118.2 (7)	C58—C57—C56	117.9 (7)
C12—C11—C15	121.1 (8)	C58—C57—C61	121.0 (8)
C10—C11—C15	120.7 (8)	C56—C57—C61	121.1 (8)
C11—C12—C13	122.5 (8)	C57—C58—C59	122.5 (7)
C11—C12—H4	118.7	C57—C58—H41	118.8
C13—C12—H4	118.7	C59—C58—H41	118.8
C12—C13—C8	118.0 (7)	C58—C59—C54	119.1 (7)
C12—C13—C16	118.6 (7)	C58—C59—C62	118.8 (7)
C8—C13—C16	123.5 (7)	C54—C59—C62	122.1 (7)
C9—C14—H5	109.5	C55—C60—H42	109.5
C9—C14—H6	109.5	C55—C60—H43	109.5
H5—C14—H6	109.5	H42—C60—H43	109.5
C9—C14—H7	109.5	C55—C60—H44	109.5
H5—C14—H7	109.5	H42—C60—H44	109.5
H6—C14—H7	109.5	H43—C60—H44	109.5
C11—C15—H8	109.5	C57—C61—H45	109.5
C11—C15—H9	109.5	C57—C61—H46	109.5
H8—C15—H9	109.5	H45—C61—H46	109.5
C11—C15—H10	109.5	C57—C61—H47	109.5
H8—C15—H10	109.5	H45—C61—H47	109.5
H9—C15—H10	109.5	H46—C61—H47	109.5
C13—C16—H11	109.5	C59—C62—H48	109.5
C13—C16—H12	109.5	C59—C62—H49	109.5
H11—C16—H12	109.5	H48—C62—H49	109.5
C13—C16—H13	109.5	C59—C62—H50	109.5
H11—C16—H13	109.5	H48—C62—H50	109.5
H12—C16—H13	109.5	H49—C62—H50	109.5
C18—C17—C22	119.1 (7)	C64—C63—C68	119.0 (7)
C18—C17—P2	119.5 (5)	C64—C63—P5	119.7 (6)
C22—C17—P2	121.1 (6)	C68—C63—P5	120.9 (6)
C19—C18—C17	119.7 (7)	C63—C64—C65	119.8 (7)
C19—C18—H14	120.1	C63—C64—H51	120.1
C17—C18—H14	120.1	C65—C64—H51	120.1
C20—C19—C18	120.9 (8)	C66—C65—C64	120.3 (8)
C20—C19—H15	119.6	C66—C65—H52	119.9
C18—C19—H15	119.6	C64—C65—H52	119.9
C19—C20—C21	119.8 (7)	C65—C66—C67	120.2 (8)
C19—C20—H16	120.1	C65—C66—H53	119.9
C21—C20—H16	120.1	C67—C66—H53	119.9
C20—C21—C22	120.0 (7)	C68—C67—C66	120.1 (8)
C20—C21—H17	120.0	C68—C67—H54	120.0
C22—C21—H17	120.0	C66—C67—H54	120.0
C21—C22—C17	120.4 (7)	C67—C68—C63	120.5 (8)
C21—C22—H18	119.8	C67—C68—H55	119.7
C17—C22—H18	119.8	C63—C68—H55	119.7
C28—C23—C24	119.1 (7)	C74—C69—C70	118.8 (7)
C28—C23—P2	118.6 (6)	C74—C69—P5	119.0 (6)
C24—C23—P2	121.8 (5)	C70—C69—P5	122.0 (6)
C25—C24—C23	118.8 (7)	C71—C70—C69	119.3 (8)
C25—C24—H19	120.6	C71—C70—H56	120.3
C23—C24—H19	120.6	C69—C70—H56	120.3
C26—C25—C24	121.8 (8)	C72—C71—C70	121.0 (8)
C26—C25—H20	119.1	C72—C71—H57	119.5
C24—C25—H20	119.1	C70—C71—H57	119.5
C25—C26—C27	119.6 (7)	C71—C72—C73	120.4 (7)
C25—C26—H21	120.2	C71—C72—H58	119.8
C27—C26—H21	120.2	C73—C72—H58	119.8
C26—C27—C28	119.4 (7)	C74—C73—C72	118.1 (8)
C26—C27—H22	120.3	C74—C73—H59	120.9
C28—C27—H22	120.3	C72—C73—H59	120.9
C23—C28—C27	121.3 (7)	C69—C74—C73	122.4 (8)
C23—C28—H23	119.4	C69—C74—H60	118.8
C27—C28—H23	119.4	C73—C74—H60	118.8
C30—C29—C34	119.5 (7)	C76—C75—C80	120.3 (7)
C30—C29—P2	122.5 (6)	C76—C75—P5	121.8 (6)
C34—C29—P2	117.9 (5)	C80—C75—P5	117.8 (6)
C31—C30—C29	121.3 (7)	C77—C76—C75	119.5 (8)
C31—C30—H24	119.4	C77—C76—H61	120.3
C29—C30—H24	119.4	C75—C76—H61	120.3
C30—C31—C32	118.9 (8)	C78—C77—C76	120.5 (8)
C30—C31—H25	120.5	C78—C77—H62	119.8
C32—C31—H25	120.5	C76—C77—H62	119.8
C33—C32—C31	120.5 (7)	C79—C78—C77	120.8 (8)
C33—C32—H26	119.7	C79—C78—H63	119.6
C31—C32—H26	119.7	C77—C78—H63	119.6
C32—C33—C34	120.9 (7)	C78—C79—C80	120.2 (8)
C32—C33—H27	119.6	C78—C79—H64	119.9
C34—C33—H27	119.6	C80—C79—H64	119.9
C33—C34—C29	118.8 (7)	C75—C80—C79	118.7 (7)
C33—C34—P3	123.7 (6)	C75—C80—P6	116.8 (6)
C29—C34—P3	117.0 (5)	C79—C80—P6	124.4 (6)
C36—C35—C40	118.7 (7)	C86—C81—C82	119.0 (7)
C36—C35—P3	119.7 (6)	C86—C81—P6	123.4 (6)
C40—C35—P3	121.6 (6)	C82—C81—P6	117.5 (6)
C35—C36—C37	120.3 (8)	C83—C82—C81	119.6 (8)
C35—C36—H28	119.8	C83—C82—H65	120.2
C37—C36—H28	119.8	C81—C82—H65	120.2
C38—C37—C36	120.6 (8)	C84—C83—C82	120.5 (8)
C38—C37—H29	119.7	C84—C83—H66	119.7
C36—C37—H29	119.7	C82—C83—H66	119.7
C39—C38—C37	119.2 (8)	C83—C84—C85	120.5 (8)
C39—C38—H30	120.4	C83—C84—H67	119.7
C37—C38—H30	120.4	C85—C84—H67	119.7
C38—C39—C40	121.6 (8)	C86—C85—C84	119.6 (9)
C38—C39—H31	119.2	C86—C85—H68	120.2
C40—C39—H31	119.2	C84—C85—H68	120.2
C39—C40—C35	119.6 (8)	C81—C86—C85	120.7 (8)
C39—C40—H32	120.2	C81—C86—H69	119.6
C35—C40—H32	120.2	C85—C86—H69	119.6
C46—C41—C42	119.2 (7)	C92—C87—C88	118.5 (7)
C46—C41—P3	122.8 (6)	C92—C87—P6	122.4 (6)
C42—C41—P3	117.8 (6)	C88—C87—P6	118.9 (6)
C43—C42—C41	120.2 (8)	C89—C88—C87	121.5 (8)
C43—C42—H33	119.9	C89—C88—H70	119.3
C41—C42—H33	119.9	C87—C88—H70	119.3
C42—C43—C44	120.6 (8)	C88—C89—C90	119.6 (8)
C42—C43—H34	119.7	C88—C89—H71	120.2
C44—C43—H34	119.7	C90—C89—H71	120.2
C43—C44—C45	120.3 (7)	C91—C90—C89	119.5 (8)
C43—C44—H35	119.9	C91—C90—H72	120.2
C45—C44—H35	119.9	C89—C90—H72	120.2
C44—C45—C46	119.4 (8)	C90—C91—C92	120.5 (8)
C44—C45—H36	120.3	C90—C91—H73	119.7
C46—C45—H36	120.3	C92—C91—H73	119.7
C41—C46—C45	120.3 (7)	C91—C92—C87	120.3 (8)
C41—C46—H37	119.8	C91—C92—H74	119.8
C45—C46—H37	119.8	C87—C92—H74	119.8
			
C1—Ru1—Ru2—C3	−104.0 (5)	C47—Ru4—Ru5—C50	12.6 (3)
P2—Ru1—Ru2—C3	−9.8 (5)	P5—Ru4—Ru5—C50	106.0 (3)
P1—Ru1—Ru2—C3	−8.3 (4)	P6—Ru4—Ru5—C50	−86.1 (2)
P3—Ru1—Ru2—C3	156.7 (4)	P4—Ru4—Ru5—C50	108.3 (2)
Ru3—Ru1—Ru2—C3	54.4 (4)	Ru6—Ru4—Ru5—C50	171.2 (2)
C1—Ru1—Ru2—C4	12.6 (3)	C47—Ru4—Ru5—C49	−105.3 (5)
P2—Ru1—Ru2—C4	106.8 (3)	P5—Ru4—Ru5—C49	−12.0 (5)
P1—Ru1—Ru2—C4	108.3 (2)	P6—Ru4—Ru5—C49	156.0 (4)
P3—Ru1—Ru2—C4	−86.6 (2)	P4—Ru4—Ru5—C49	−9.7 (4)
Ru3—Ru1—Ru2—C4	171.0 (2)	Ru6—Ru4—Ru5—C49	53.2 (4)
C1—Ru1—Ru2—C2	117.2 (3)	C47—Ru4—Ru5—C48	116.8 (3)
P2—Ru1—Ru2—C2	−148.7 (3)	P5—Ru4—Ru5—C48	−149.9 (3)
P1—Ru1—Ru2—C2	−147.1 (2)	P6—Ru4—Ru5—C48	18.0 (2)
P3—Ru1—Ru2—C2	17.9 (2)	P4—Ru4—Ru5—C48	−147.6 (2)
Ru3—Ru1—Ru2—C2	−84.5 (2)	Ru6—Ru4—Ru5—C48	−84.7 (2)
C1—Ru1—Ru2—P1	−95.7 (2)	C47—Ru4—Ru5—P4	−95.6 (2)
P2—Ru1—Ru2—P1	−1.57 (14)	P5—Ru4—Ru5—P4	−2.30 (15)
P3—Ru1—Ru2—P1	165.02 (8)	P6—Ru4—Ru5—P4	165.66 (8)
Ru3—Ru1—Ru2—P1	62.64 (6)	Ru6—Ru4—Ru5—P4	62.90 (6)
C1—Ru1—Ru2—Ru3	−158.4 (2)	C47—Ru4—Ru5—Ru6	−158.5 (2)
P2—Ru1—Ru2—Ru3	−64.21 (14)	P5—Ru4—Ru5—Ru6	−65.19 (15)
P1—Ru1—Ru2—Ru3	−62.64 (6)	P6—Ru4—Ru5—Ru6	102.76 (6)
P3—Ru1—Ru2—Ru3	102.38 (6)	P4—Ru4—Ru5—Ru6	−62.90 (6)
C3—Ru2—Ru3—C6	−0.7 (3)	C50—Ru5—Ru6—C52	128.6 (7)
C4—Ru2—Ru3—C6	126.3 (8)	C49—Ru5—Ru6—C52	0.5 (3)
C2—Ru2—Ru3—C6	−97.4 (3)	C48—Ru5—Ru6—C52	−95.8 (3)
P1—Ru2—Ru3—C6	94.5 (2)	P4—Ru5—Ru6—C52	96.3 (2)
Ru1—Ru2—Ru3—C6	154.2 (2)	Ru4—Ru5—Ru6—C52	155.9 (2)
C3—Ru2—Ru3—C7	−104.1 (10)	C50—Ru5—Ru6—C53	28.6 (12)
C4—Ru2—Ru3—C7	22.9 (13)	C49—Ru5—Ru6—C53	−99.5 (10)
C2—Ru2—Ru3—C7	159.1 (10)	C48—Ru5—Ru6—C53	164.2 (10)
P1—Ru2—Ru3—C7	−8.9 (10)	P4—Ru5—Ru6—C53	−3.7 (10)
Ru1—Ru2—Ru3—C7	50.7 (10)	Ru4—Ru5—Ru6—C53	55.9 (10)
C3—Ru2—Ru3—C5	94.6 (3)	C50—Ru5—Ru6—C51	−136.0 (7)
C4—Ru2—Ru3—C5	−138.4 (8)	C49—Ru5—Ru6—C51	95.8 (3)
C2—Ru2—Ru3—C5	−2.1 (3)	C48—Ru5—Ru6—C51	−0.5 (3)
P1—Ru2—Ru3—C5	−170.2 (2)	P4—Ru5—Ru6—C51	−168.3 (2)
Ru1—Ru2—Ru3—C5	−110.5 (2)	Ru4—Ru5—Ru6—C51	−108.7 (2)
C3—Ru2—Ru3—P1	−95.2 (2)	C50—Ru5—Ru6—P4	32.3 (7)
C4—Ru2—Ru3—P1	31.8 (8)	C49—Ru5—Ru6—P4	−95.8 (2)
C2—Ru2—Ru3—P1	168.0 (2)	C48—Ru5—Ru6—P4	167.9 (2)
Ru1—Ru2—Ru3—P1	59.64 (6)	Ru4—Ru5—Ru6—P4	59.61 (6)
C3—Ru2—Ru3—Ru1	−154.8 (2)	C50—Ru5—Ru6—Ru4	−27.3 (7)
C4—Ru2—Ru3—Ru1	−27.8 (7)	C49—Ru5—Ru6—Ru4	−155.4 (2)
C2—Ru2—Ru3—Ru1	108.4 (2)	C48—Ru5—Ru6—Ru4	108.3 (2)
P1—Ru2—Ru3—Ru1	−59.64 (6)	P4—Ru5—Ru6—Ru4	−59.61 (6)
C1—Ru1—Ru3—C6	−8.1 (6)	C47—Ru4—Ru6—C52	−5.7 (6)
P2—Ru1—Ru3—C6	111.5 (4)	P5—Ru4—Ru6—C52	114.9 (4)
P1—Ru1—Ru3—C6	18.1 (4)	P6—Ru4—Ru6—C52	−151.5 (4)
P3—Ru1—Ru3—C6	−154.8 (4)	P4—Ru4—Ru6—C52	21.5 (4)
Ru2—Ru1—Ru3—C6	−47.0 (4)	Ru5—Ru4—Ru6—C52	−43.9 (4)
C1—Ru1—Ru3—C7	−130.4 (5)	C47—Ru4—Ru6—C53	−130.2 (5)
P2—Ru1—Ru3—C7	−10.8 (3)	P5—Ru4—Ru6—C53	−9.6 (3)
P1—Ru1—Ru3—C7	−104.2 (3)	P6—Ru4—Ru6—C53	84.1 (3)
P3—Ru1—Ru3—C7	82.9 (3)	P4—Ru4—Ru6—C53	−102.9 (3)
Ru2—Ru1—Ru3—C7	−169.3 (3)	Ru5—Ru4—Ru6—C53	−168.4 (3)
C1—Ru1—Ru3—C5	120.8 (5)	C47—Ru4—Ru6—C51	122.2 (5)
P2—Ru1—Ru3—C5	−119.6 (2)	P5—Ru4—Ru6—C51	−117.2 (2)
P1—Ru1—Ru3—C5	147.0 (2)	P6—Ru4—Ru6—C51	−23.6 (2)
P3—Ru1—Ru3—C5	−25.9 (2)	P4—Ru4—Ru6—C51	149.4 (3)
Ru2—Ru1—Ru3—C5	81.9 (2)	Ru5—Ru4—Ru6—C51	84.0 (2)
C1—Ru1—Ru3—P1	−26.2 (4)	C47—Ru4—Ru6—P4	−27.2 (4)
P2—Ru1—Ru3—P1	93.41 (8)	P5—Ru4—Ru6—P4	93.37 (8)
P3—Ru1—Ru3—P1	−172.90 (8)	P6—Ru4—Ru6—P4	−172.97 (8)
Ru2—Ru1—Ru3—P1	−65.09 (6)	Ru5—Ru4—Ru6—P4	−65.43 (6)
C1—Ru1—Ru3—Ru2	38.9 (4)	C47—Ru4—Ru6—Ru5	38.2 (4)
P2—Ru1—Ru3—Ru2	158.50 (5)	P5—Ru4—Ru6—Ru5	158.80 (6)
P1—Ru1—Ru3—Ru2	65.09 (6)	P6—Ru4—Ru6—Ru5	−107.54 (6)
P3—Ru1—Ru3—Ru2	−107.81 (6)	P4—Ru4—Ru6—Ru5	65.43 (6)
C1—Ru1—P1—C8	−52.8 (4)	C47—Ru4—P4—C54	−52.5 (4)
P2—Ru1—P1—C8	36.8 (4)	P5—Ru4—P4—C54	37.3 (3)
P3—Ru1—P1—C8	165.1 (4)	P6—Ru4—P4—C54	166.7 (3)
Ru2—Ru1—P1—C8	−142.6 (4)	Ru5—Ru4—P4—C54	−141.8 (3)
Ru3—Ru1—P1—C8	142.2 (4)	Ru6—Ru4—P4—C54	143.4 (3)
C1—Ru1—P1—Ru3	164.9 (2)	C47—Ru4—P4—Ru6	164.2 (2)
P2—Ru1—P1—Ru3	−105.42 (6)	P5—Ru4—P4—Ru6	−106.02 (7)
P3—Ru1—P1—Ru3	22.9 (3)	P6—Ru4—P4—Ru6	23.3 (3)
Ru2—Ru1—P1—Ru3	75.20 (5)	Ru5—Ru4—P4—Ru6	74.86 (5)
C1—Ru1—P1—Ru2	89.7 (2)	C47—Ru4—P4—Ru5	89.3 (2)
P2—Ru1—P1—Ru2	179.38 (6)	P5—Ru4—P4—Ru5	179.12 (6)
P3—Ru1—P1—Ru2	−52.3 (3)	P6—Ru4—P4—Ru5	−51.6 (3)
Ru3—Ru1—P1—Ru2	−75.20 (5)	Ru6—Ru4—P4—Ru5	−74.86 (5)
C6—Ru3—P1—C8	55.0 (4)	C52—Ru6—P4—C54	57.3 (4)
C7—Ru3—P1—C8	−37.9 (4)	C53—Ru6—P4—C54	−36.0 (4)
C5—Ru3—P1—C8	161.4 (5)	C51—Ru6—P4—C54	165.9 (5)
Ru2—Ru3—P1—C8	144.3 (4)	Ru5—Ru6—P4—C54	144.9 (4)
Ru1—Ru3—P1—C8	−135.7 (4)	Ru4—Ru6—P4—C54	−135.2 (4)
C6—Ru3—P1—Ru1	−169.3 (2)	C52—Ru6—P4—Ru4	−167.5 (2)
C7—Ru3—P1—Ru1	97.7 (3)	C53—Ru6—P4—Ru4	99.3 (3)
C5—Ru3—P1—Ru1	−62.9 (4)	C51—Ru6—P4—Ru4	−58.9 (4)
Ru2—Ru3—P1—Ru1	−80.07 (5)	Ru5—Ru6—P4—Ru4	−79.84 (5)
C6—Ru3—P1—Ru2	−89.2 (2)	C52—Ru6—P4—Ru5	−87.7 (2)
C7—Ru3—P1—Ru2	177.8 (2)	C53—Ru6—P4—Ru5	179.1 (2)
C5—Ru3—P1—Ru2	17.2 (4)	C51—Ru6—P4—Ru5	20.9 (4)
Ru1—Ru3—P1—Ru2	80.07 (5)	Ru4—Ru6—P4—Ru5	79.84 (5)
C3—Ru2—P1—C8	−50.5 (4)	C50—Ru5—P4—C54	46.5 (4)
C4—Ru2—P1—C8	48.1 (5)	C49—Ru5—P4—C54	−52.8 (4)
C2—Ru2—P1—C8	−163.4 (5)	C48—Ru5—P4—C54	−165.7 (5)
Ru3—Ru2—P1—C8	−142.6 (4)	Ru6—Ru5—P4—C54	−144.3 (4)
Ru1—Ru2—P1—C8	133.8 (4)	Ru4—Ru5—P4—C54	132.2 (4)
C3—Ru2—P1—Ru1	175.7 (2)	C50—Ru5—P4—Ru4	−85.7 (3)
C4—Ru2—P1—Ru1	−85.7 (3)	C49—Ru5—P4—Ru4	175.0 (2)
C2—Ru2—P1—Ru1	62.8 (4)	C48—Ru5—P4—Ru4	62.1 (4)
Ru3—Ru2—P1—Ru1	83.62 (5)	Ru6—Ru5—P4—Ru4	83.46 (5)
C3—Ru2—P1—Ru3	92.0 (2)	C50—Ru5—P4—Ru6	−169.2 (3)
C4—Ru2—P1—Ru3	−169.3 (3)	C49—Ru5—P4—Ru6	91.5 (2)
C2—Ru2—P1—Ru3	−20.9 (4)	C48—Ru5—P4—Ru6	−21.3 (4)
Ru1—Ru2—P1—Ru3	−83.62 (5)	Ru4—Ru5—P4—Ru6	−83.46 (5)
C1—Ru1—P2—C17	38.4 (4)	C47—Ru4—P5—C63	38.4 (4)
P1—Ru1—P2—C17	−58.3 (3)	P6—Ru4—P5—C63	134.7 (3)
P3—Ru1—P2—C17	134.9 (3)	P4—Ru4—P5—C63	−57.9 (3)
Ru2—Ru1—P2—C17	−57.0 (3)	Ru5—Ru4—P5—C63	−56.0 (3)
Ru3—Ru1—P2—C17	−111.0 (3)	Ru6—Ru4—P5—C63	−111.0 (3)
C1—Ru1—P2—C23	169.3 (4)	C47—Ru4—P5—C69	168.9 (4)
P1—Ru1—P2—C23	72.6 (3)	P6—Ru4—P5—C69	−94.8 (3)
P3—Ru1—P2—C23	−94.2 (3)	P4—Ru4—P5—C69	72.6 (3)
Ru2—Ru1—P2—C23	73.8 (3)	Ru5—Ru4—P5—C69	74.5 (3)
Ru3—Ru1—P2—C23	19.9 (3)	Ru6—Ru4—P5—C69	19.5 (3)
C1—Ru1—P2—C29	−77.3 (3)	C47—Ru4—P5—C75	−75.5 (3)
P1—Ru1—P2—C29	−174.1 (2)	P6—Ru4—P5—C75	20.8 (3)
P3—Ru1—P2—C29	19.2 (2)	P4—Ru4—P5—C75	−171.8 (3)
Ru2—Ru1—P2—C29	−172.8 (2)	Ru5—Ru4—P5—C75	−169.9 (3)
Ru3—Ru1—P2—C29	133.3 (2)	Ru6—Ru4—P5—C75	135.1 (3)
C1—Ru1—P3—C34	66.0 (3)	C47—Ru4—P6—C80	64.0 (3)
P2—Ru1—P3—C34	−20.9 (2)	P5—Ru4—P6—C80	−23.2 (3)
P1—Ru1—P3—C34	−151.8 (3)	P4—Ru4—P6—C80	−155.0 (3)
Ru2—Ru1—P3—C34	164.1 (2)	Ru5—Ru4—P6—C80	161.3 (2)
Ru3—Ru1—P3—C34	−132.8 (2)	Ru6—Ru4—P6—C80	−135.5 (2)
C1—Ru1—P3—C41	−179.9 (4)	C47—Ru4—P6—C87	178.0 (4)
P2—Ru1—P3—C41	93.2 (3)	P5—Ru4—P6—C87	90.9 (3)
P1—Ru1—P3—C41	−37.7 (4)	P4—Ru4—P6—C87	−41.0 (4)
Ru2—Ru1—P3—C41	−81.8 (3)	Ru5—Ru4—P6—C87	−84.7 (3)
Ru3—Ru1—P3—C41	−18.7 (3)	Ru6—Ru4—P6—C87	−21.5 (3)
C1—Ru1—P3—C35	−52.7 (4)	C47—Ru4—P6—C81	−56.2 (4)
P2—Ru1—P3—C35	−139.6 (3)	P5—Ru4—P6—C81	−143.4 (3)
P1—Ru1—P3—C35	89.5 (4)	P4—Ru4—P6—C81	84.7 (4)
Ru2—Ru1—P3—C35	45.4 (3)	Ru5—Ru4—P6—C81	41.0 (3)
Ru3—Ru1—P3—C35	108.5 (3)	Ru6—Ru4—P6—C81	104.2 (3)
Ru1—P1—C8—C9	−88.1 (6)	Ru4—P4—C54—C55	−89.1 (6)
Ru3—P1—C8—C9	31.6 (8)	Ru6—P4—C54—C55	30.8 (8)
Ru2—P1—C8—C9	154.7 (4)	Ru5—P4—C54—C55	155.4 (5)
Ru1—P1—C8—C13	89.2 (6)	Ru4—P4—C54—C59	87.6 (6)
Ru3—P1—C8—C13	−151.1 (5)	Ru6—P4—C54—C59	−152.5 (5)
Ru2—P1—C8—C13	−28.0 (8)	Ru5—P4—C54—C59	−27.8 (8)
C13—C8—C9—C10	−3.2 (11)	C59—C54—C55—C56	−2.3 (11)
P1—C8—C9—C10	174.1 (6)	P4—C54—C55—C56	174.5 (6)
C13—C8—C9—C14	178.4 (7)	C59—C54—C55—C60	178.7 (7)
P1—C8—C9—C14	−4.3 (10)	P4—C54—C55—C60	−4.4 (10)
C8—C9—C10—C11	0.9 (11)	C54—C55—C56—C57	0.3 (12)
C14—C9—C10—C11	179.4 (7)	C60—C55—C56—C57	179.3 (7)
C9—C10—C11—C12	1.3 (11)	C55—C56—C57—C58	2.0 (12)
C9—C10—C11—C15	−177.7 (7)	C55—C56—C57—C61	−177.0 (8)
C10—C11—C12—C13	−1.1 (11)	C56—C57—C58—C59	−2.2 (12)
C15—C11—C12—C13	177.8 (7)	C61—C57—C58—C59	176.8 (7)
C11—C12—C13—C8	−1.1 (11)	C57—C58—C59—C54	0.2 (12)
C11—C12—C13—C16	179.5 (7)	C57—C58—C59—C62	179.8 (7)
C9—C8—C13—C12	3.3 (10)	C55—C54—C59—C58	2.1 (11)
P1—C8—C13—C12	−174.0 (5)	P4—C54—C59—C58	−174.7 (6)
C9—C8—C13—C16	−177.4 (7)	C55—C54—C59—C62	−177.6 (7)
P1—C8—C13—C16	5.3 (10)	P4—C54—C59—C62	5.7 (10)
C23—P2—C17—C18	−46.7 (7)	C69—P5—C63—C64	−46.0 (7)
C29—P2—C17—C18	−150.8 (6)	C75—P5—C63—C64	−151.2 (6)
Ru1—P2—C17—C18	91.4 (6)	Ru4—P5—C63—C64	92.4 (6)
C23—P2—C17—C22	138.4 (6)	C69—P5—C63—C68	140.6 (6)
C29—P2—C17—C22	34.4 (7)	C75—P5—C63—C68	35.3 (7)
Ru1—P2—C17—C22	−83.5 (6)	Ru4—P5—C63—C68	−81.0 (6)
C22—C17—C18—C19	−1.1 (12)	C68—C63—C64—C65	−0.9 (11)
P2—C17—C18—C19	−176.0 (6)	P5—C63—C64—C65	−174.5 (6)
C17—C18—C19—C20	0.7 (12)	C63—C64—C65—C66	−0.2 (12)
C18—C19—C20—C21	−0.9 (13)	C64—C65—C66—C67	0.4 (12)
C19—C20—C21—C22	1.5 (13)	C65—C66—C67—C68	0.6 (13)
C20—C21—C22—C17	−2.0 (12)	C66—C67—C68—C63	−1.7 (12)
C18—C17—C22—C21	1.8 (12)	C64—C63—C68—C67	1.9 (12)
P2—C17—C22—C21	176.6 (6)	P5—C63—C68—C67	175.3 (6)
C17—P2—C23—C28	166.0 (6)	C63—P5—C69—C74	160.6 (6)
C29—P2—C23—C28	−86.8 (6)	C75—P5—C69—C74	−92.9 (7)
Ru1—P2—C23—C28	30.6 (7)	Ru4—P5—C69—C74	25.7 (8)
C17—P2—C23—C24	−21.2 (7)	C63—P5—C69—C70	−24.7 (7)
C29—P2—C23—C24	85.9 (6)	C75—P5—C69—C70	81.8 (7)
Ru1—P2—C23—C24	−156.6 (5)	Ru4—P5—C69—C70	−159.6 (5)
C28—C23—C24—C25	−1.4 (11)	C74—C69—C70—C71	−1.8 (12)
P2—C23—C24—C25	−174.1 (6)	P5—C69—C70—C71	−176.6 (6)
C23—C24—C25—C26	1.4 (12)	C69—C70—C71—C72	1.7 (13)
C24—C25—C26—C27	−0.4 (13)	C70—C71—C72—C73	−0.5 (13)
C25—C26—C27—C28	−0.7 (12)	C71—C72—C73—C74	−0.6 (13)
C24—C23—C28—C27	0.3 (12)	C70—C69—C74—C73	0.8 (12)
P2—C23—C28—C27	173.3 (6)	P5—C69—C74—C73	175.6 (6)
C26—C27—C28—C23	0.8 (12)	C72—C73—C74—C69	0.4 (13)
C17—P2—C29—C30	44.8 (7)	C63—P5—C75—C76	45.1 (7)
C23—P2—C29—C30	−63.8 (7)	C69—P5—C75—C76	−63.2 (7)
Ru1—P2—C29—C30	168.6 (6)	Ru4—P5—C75—C76	167.6 (6)
C17—P2—C29—C34	−137.8 (6)	C63—P5—C75—C80	−137.2 (6)
C23—P2—C29—C34	113.7 (6)	C69—P5—C75—C80	114.5 (6)
Ru1—P2—C29—C34	−13.9 (6)	Ru4—P5—C75—C80	−14.7 (6)
C34—C29—C30—C31	−1.7 (12)	C80—C75—C76—C77	3.0 (13)
P2—C29—C30—C31	175.8 (7)	P5—C75—C76—C77	−179.4 (7)
C29—C30—C31—C32	2.4 (13)	C75—C76—C77—C78	−1.2 (14)
C30—C31—C32—C33	−2.9 (13)	C76—C77—C78—C79	−0.2 (15)
C31—C32—C33—C34	2.6 (12)	C77—C78—C79—C80	−0.3 (14)
C32—C33—C34—C29	−1.8 (11)	C76—C75—C80—C79	−3.4 (11)
C32—C33—C34—P3	−173.9 (6)	P5—C75—C80—C79	178.8 (6)
C30—C29—C34—C33	1.3 (11)	C76—C75—C80—P6	173.1 (6)
P2—C29—C34—C33	−176.2 (6)	P5—C75—C80—P6	−4.6 (8)
C30—C29—C34—P3	173.9 (6)	C78—C79—C80—C75	2.1 (12)
P2—C29—C34—P3	−3.6 (8)	C78—C79—C80—P6	−174.1 (7)
C41—P3—C34—C33	69.1 (7)	C87—P6—C80—C75	−101.2 (6)
C35—P3—C34—C33	−39.2 (7)	C81—P6—C80—C75	152.6 (6)
Ru1—P3—C34—C33	−168.4 (6)	Ru4—P6—C80—C75	21.8 (6)
C41—P3—C34—C29	−103.1 (6)	C87—P6—C80—C79	75.1 (7)
C35—P3—C34—C29	148.6 (6)	C81—P6—C80—C79	−31.1 (7)
Ru1—P3—C34—C29	19.4 (6)	Ru4—P6—C80—C79	−161.8 (6)
C34—P3—C35—C36	−88.0 (7)	C80—P6—C81—C86	81.7 (7)
C41—P3—C35—C36	164.9 (6)	C87—P6—C81—C86	−24.5 (7)
Ru1—P3—C35—C36	32.7 (7)	Ru4—P6—C81—C86	−156.4 (6)
C34—P3—C35—C40	90.3 (6)	C80—P6—C81—C82	−95.2 (6)
C41—P3—C35—C40	−16.7 (7)	C87—P6—C81—C82	158.5 (6)
Ru1—P3—C35—C40	−148.9 (5)	Ru4—P6—C81—C82	26.6 (7)
C40—C35—C36—C37	−0.4 (12)	C86—C81—C82—C83	−0.7 (12)
P3—C35—C36—C37	178.0 (7)	P6—C81—C82—C83	176.4 (6)
C35—C36—C37—C38	0.9 (13)	C81—C82—C83—C84	−0.1 (13)
C36—C37—C38—C39	0.1 (13)	C82—C83—C84—C85	1.0 (13)
C37—C38—C39—C40	−1.6 (13)	C83—C84—C85—C86	−1.0 (13)
C38—C39—C40—C35	2.1 (12)	C82—C81—C86—C85	0.6 (12)
C36—C35—C40—C39	−1.1 (11)	P6—C81—C86—C85	−176.2 (6)
P3—C35—C40—C39	−179.4 (6)	C84—C85—C86—C81	0.2 (13)
C34—P3—C41—C46	8.9 (7)	C80—P6—C87—C92	15.4 (7)
C35—P3—C41—C46	116.6 (7)	C81—P6—C87—C92	123.3 (7)
Ru1—P3—C41—C46	−108.2 (6)	Ru4—P6—C87—C92	−101.6 (7)
C34—P3—C41—C42	−176.2 (6)	C80—P6—C87—C88	−169.0 (6)
C35—P3—C41—C42	−68.4 (6)	C81—P6—C87—C88	−61.1 (7)
Ru1—P3—C41—C42	66.8 (6)	Ru4—P6—C87—C88	73.9 (6)
C46—C41—C42—C43	0.5 (11)	C92—C87—C88—C89	−0.2 (12)
P3—C41—C42—C43	−174.7 (6)	P6—C87—C88—C89	−176.0 (6)
C41—C42—C43—C44	1.4 (12)	C87—C88—C89—C90	−0.2 (12)
C42—C43—C44—C45	−2.0 (12)	C88—C89—C90—C91	1.1 (13)
C43—C44—C45—C46	0.7 (12)	C89—C90—C91—C92	−1.6 (14)
C42—C41—C46—C45	−1.7 (12)	C90—C91—C92—C87	1.1 (14)
P3—C41—C46—C45	173.2 (6)	C88—C87—C92—C91	−0.2 (12)
C44—C45—C46—C41	1.2 (13)	P6—C87—C92—C91	175.4 (7)
